# Dry-Cupping as an Excito-Secretory Remedial Agent

**Published:** 1864-12

**Authors:** B. H. Washington

**Affiliations:** Acting Surgeon.


### CONFEDERATE STATES MEDICAL & SURGICAL JOURNAL.

VOL. I. RICHMOND, DECEMBER, 1864. No. 12.

### ORIGINAL COMMUNICATIONS.

ART. I.—*Dry-Cupping as an Excito-Secretory Remedial Agent.* By B. H. WASHINGTON, Acting Surgeon.

About thirty years ago, Dr. J. V. Prather, subsequently Professor of Surgery in the St. Louis Medical University, came to the conclusion that he could control the secretions from all the various organs of the system by dry-cupping on the spinal column, and having permanently added that remedial agent to his armamentarium, wrought many surprising cures. He found, however, that a great metaphysician spoke truly, when he said, "high truths, like high mountains, are apt to veil themselves in clouds," and as the admirable work of Dr. H. F. Campbell had not dispelled the clouds then surrounding the excito-secretory system, Dr. Prather could not give a satisfactory rationale for his procedure, but could only dogmatically affirm that he could control the secretions by dry-cupping. His medical confreres were not content to take his affirmation, but laughed immoderately and often at "Prather's hobby," and paid comparatively little attention to the subject. Having been duly instructed by Dr. P., while attending the lectures in St. Louis, we have practised as he did, for nearly twenty years, (though not actively engaged all that time in the practice), and our contributions to the medical journals, recommending dry-cupping, have met with far more favor than we had any reason to anticipate.

Now, however, since Dr. Campbell has dispelled the clouds surrounding the excito-secretory system, we have concluded to present dry-cupping as it truly is, a most powerful excito-secretory remedial agent, humbly hoping for a more favorable and general assent to the correctness of our propositions than that hitherto accorded us. We shall first present the cases, abridged as much as practicable, and then the logical deductions to be drawn from them, guided by the expositions of Dr. Campbell, and we are confident a much larger number of readers will cheerfully acquiesce in their correctness than when dogmatically presented in by-gone days.

*Case* 1.—Mrs. Y., Logan county, Ky., 1848. She had suffered for seven months from intermittent fever, though one of the best physicians in the state had been in attendance on her. We called on the physician, and having ascertained his plan of treatment, returned and used precisely the same treatment to a single grain, but added on dry-cupping, and sponging the whole surface with hot water every alternate night. This we did because we knew dry-cupping to be the most powerful tonic and alterative agent yet presented to the profession. Such a thorough cure was made that Mrs. Y. never had another chill during the four years she lived after this occurrence.

This case is not, however, introduced merely to show that obstinate cases of intermittent can be cured by dry-cupping, but for a remarkable coincident cure effected, and which was truly surprising. When we took charge of her case, Mrs. Y. also informed us that she had been afflicted with tetter on her leg for thirty years, and that many physicians had prescribed for her in vain. As this was the first case we had in that neighborhood, and we were anxious not to have an unfortunate result, we concluded to stave off the treatment of the tetter, for we had no idea a cure could be effected. We, therefore, told her we would wait until she was cured of the chills before we did anything for the tetter, and determined to examine the medical journals and late works in the meanwhile, to ascertain whether we could find anything not likely to have been tried by any one of the aforesaid physicians. We looked over the journals in vain; not a thing could we find presenting any probability of effecting a cure. Time rolled on, and as Mrs. Y. said nothing to us concerning the tetter, we took good care not to allude to it to her. Some five or six months afterwards, we heard her boasting of her fine health: "Yes," said we, "and you owe that to dry-cupping." "Well, then," replied she, "it must be the dry-cupping that has cured my leg, for it is healed over as smoothly as the other." This was joyful news to us, and we determined to profit by it.

The dry-cupping in this case was continued about four months.

*Case* 2.—The next day after the above mentioned conversation with Mrs. Y., we determined to ascertain with certainty whether the cure of the tetter was merely a remarkable coincidence, or whether it was a legitimate result of the dry-cupping; so we rode over to Mrs. H , a neighbor, living some two miles distant, who had been troubled with tetter on her hands for twenty years. We related to her how Mrs. Y. had been cured, but found her much like some of Dr. Prather's confreres, so hard-headed we could beat no faith in the remedy into her at all. She, however, promised to try the dry-cupping faithfully, if we would get up a liniment for rubbing on her hands. To this we readily assented, determining to furnish her with something that would do neither good nor harm; we accordingly colored some lard and carried it to her, and having instructed her daughter how to dry-cup her, awaited the result with anxiety. After about four or five months dry-cupping and the use of the colored lard, she showed me her hands, all smoothly and softly healed over. These cases demonstrated, beyond a shadow of doubt, the truth of a proposition presented by Dr. Prather, that dry-cupping was the most powerful alterative known. These cases prepared the way for the cure of

*Case* 3.—About two months after marriage, my wife was attacked with sick headache, and informed me she had been subject to such attacks all her life. We immediately commenced dry-cupping, and kept it up faithfully for five or six months, and she never had another attack of sick headache for ten years. At the expiration of that time, having accidentally combined dinner and supper together, she ate imprudently of indigestible food, and had a severe attack next day. We again tried the dry-cupping. In the course of a few weeks she had two light attacks, but perseverance soon effected another cure, and she has had none since—now about six years.

*Case* 4.—E. P., æt. 7, daughter of Mr. H. Porter, Todd county, Kentucky, found her suffering terribly with sequelæ of scarlatina; both steam and regular practice had been tried in her case, without success, and, almost without hope, her father called us in. Pulse 125; tongue dry in the middle; a little moisture around the edges; there was an abscess on one side of her neck about the size of a turkey egg, and on the other side one about the size of a hen egg; also a large abscess on her right hand, her right arm paralysed; her right leg affected with a severe sciatic pain from the hip to the knee, and so excessively tender she could scarcely bear a sheet on it; skin dry, harsh and scaly, the usual process of desquamation having been suspended, leaving the whole surface covered with a dry, thickened epidermis; complete aphonia—lungs and abdominal viscera in fair order. She was taking stimulants under the direction of one of the best physicians in the country; directed a continuance of treatment, and directed her to be well sponged with hot water every six hours, and, as her throat appeared to be very highly inflamed, directed a cold wet bandage to be applied, with a dry one outside. Calling a few hours later, during the exacerbation of her fever, found the pulse 160, and all the symptoms aggravated. The cold bandage to her throat had afforded no relief; ordered a hot one to be applied, and also to be applied to her hand. We immediately rode off to Elkton to consult an old physician, as we had been practising but a few months, and had never read of such a case in four years' reading. We were advised to give brandy and a little quinine, and the opinion was expressed that if the pulse was 120 she could not be saved. This was very discouraging, as her pulse was 125 during the remission, and 160 during the exacerbation. Having carefully re-examined the case, we concluded to take the management into our own hands; directed the stimulants to be diminished one-half, and the interval lengthened, and gradually discontinued altogether; the sponging to be kept up; a long Cayenne pepper plaster, about three inches wide, was applied the whole length of the spinal column, as a substitute for dry-cupping, and was reapplied every night. As soon as the brandy was discontinued we gave the mistura ferri composita as a tonic. She improved very rapidly; the frequent sponging removed the dead epidermis and quickened the formation of a new, healthy skin; the abscesses matured, bursted and commenced healing rapidly, and her sciatic pain was much lessened. In about a week she had improved so much that we could dry-cup her, and this was kept up regularly, every alternate evening, during the treatment. After the process of desquamation had been completed, the sponging was directed every night instead of every six hours. At the end of three weeks she was plump and rosy, and required no further medical attendance.

*Case* 5.—While dry-cupping a very intelligent lady, on the second day after delivery, for the purpose of relieving the usual disagreeable consequences resulting from delivery, she remarked that it made the uterus contract, and examination proved her statement correct. This was a new idea decidedly, and was carefully stored away in our memory for future use. Some time afterwards we had the cups applied to our own spinal column, and while the cup was over the origin of the brachial nerve, some insect stung us on the cheek, and on attempting to raise the hand, found the arm feel very heavy, as if the nerve was partially paralyzed. Good, said we, that will be just the very thing to take off muscular resistance in cases of dislocations of the humerus, and we determined to try it the first opportunity. We did not have to wait many weeks before a suitable case presented itself. A boy about seven years of age was thrown from a horse and fractured the ulna and dislocated the humerus. Dry-cupping was tried over the origin of the brachial nerve, and having rested our right hand on the knee for a fulcrum, with the left hand the humerus was promptly and easily slid into its place, the boy scarcely making a wry face. He was dry-cupped a few times afterwards, and the arm rapidly healed.

In meditating on this case, the extension of the use of dry-cupping to overcome rigidity of the os uteri and the soft parts in cases of tedious labor, quickly presented itself. The remark of the lady concerning the contraction of the uterus was remembered. Now, said we, dry-cupping has most undoubtedly produced contraction of the uterus, and it has likewise, with equal certainty, produced relaxation in the case of the dislocated humerus: why can it not, inasmuch as contraction is the proper normal function of the fundus uteri, and relaxation and expansion the normal function of the os uteri and the adjacent soft parts, produce contraction of the fundus uteri and relaxation of the os uteri at the same time. No good reason presenting itself why it should not produce the effects contemplated, it was marked down for trial. A month or two afterwards a suitable case presented itself, and upon trial it was found to answer the most sanguine expectations. The cup was first applied over the sacrum, and allowed to remain on from five to ten minutes, so as to produce complete relaxation of the os uteri and the soft parts; then another was applied higher up on the spinal column—the first cup still being kept on—and in two minutes pains came on, and the labor was easily and promptly brought to a close. Since that time we have used it with complete success in every case of tedious labor, and sometimes in cases that were not tedious, merely to shorten the sufferings of the patient. We have also used it for the purpose of causing expulsion of the placenta, when the uterus appeared too torpid to act naturally. We published an article in the *Nashville Medical and Surgical Journal*, recommending the new remedy. Some time afterwards, Professor E. D. Fenner of New Orleans, published a report of a case of tedious labor which occurred in his own practice. The lady had been in labor for thirty hours, and he had decided that it was absolutely essential to use instruments to save her life, and just as he had reached that conclusion, the remembrance of my article flashed across his mind; he immediately applied the cups as directed, and she was safely delivered in thirty minutes. Several years afterwards Dr. Hunter, of Virginia, made the same discovery, and published an article in the *Stethoscope*, recommending dry-cupping in cases of tedious labor.

*Case* 6.—In some eight or ten cases of pregnancy, where it could be conveniently applied, we have used dry-cupping steadily, for the purpose of breaking the chain of nervous sympathy, which so often brings on such serious suffering, and also for the purpose of keeping the whole secretory system in healthy action. Most remarkably beneficial results followed its use, for the patients went their full term without experiencing scarcely any of the usual distressing consequences of pregnancy. We would most cordially recommend it in all cases of pregnancy for that purpose, and that too without any apprehension of causing contraction of the uterus, for that *it will not do except the patient has reached her full term*, as will be demonstrated by the history of the next case.

*Case* 7.—We were called to see a negro woman, found her in her seventh month of pregnancy and strongly threatened with miscarriage, as labor pains had already commenced. Her owner, a physician, informed me she had miscarried six times previously, at the same period, and that he had completely exhausted his medical skill in endeavoring to prevent it. Upon examination, found the lower lumbar vertebræ and sacrum so exceedingly tender on pressure, we concluded not to try the cups first, but had a long blister applied, covering all the tender part of the spinal column, and directed a mild opiate. The blister drew finely and afforded very great relief, and as soon as the new cuticle was formed, dry-cupping was tried every alternate night during the remainder of her pregnancy, and with complete success, for she went to her full time, and was safely delivered of a fine, healthy child.

*Case* 8.—Sarah, a slave hired in our own family, aged about seventeen, subject to frequent and severe attacks of epilepsy from suppression of her catamenia. Her skin, which always appeared to be dry and torpid, became quite furfuracious for a day or two previous to an attack—tongue always loaded with fur. Had her regularly dry-cupped and sponged every alternate night, and in the course of five or six months her catamenia re-appeared in a healthy manner, and she suffered no more from epileptic convulsions during the remainder of the year. After she returned to her owner, she had one attack from extreme exposure during a snow storm, but her mother, whom we had instructed how to dry-cup with a glass tumbler, took her case in hand, and soon relieved her again; and for several years after, while we lived in that neighborhood, she had no return of convulsions, and her catamenia continued regular. We would here state, *en passant*, that we have never, at any time, used any other emmenagogue than dry-cupping and sponging the whole surface, and have uniformly found it to possess entire control over the uterine function, and it always afforded relief, whether the patient was afflicted with amenorrhea, dysmenorrhea, leucorrhea or menorrhagia. Furthermore, we would remark, that from ample experience in cases needless to mention, in cases of barrenness, a course of dry-cupping from four to six months will bring the uterine function into such a healthy condition, that conception cannot be avoided, unless the husband is utterly impotent.

In the treatment of the above case, it was necessary to use injections for several weeks to relieve the bowels, in consequence of the extreme torpor of the system, but the torpor was soon overcome. While on the subject of habitual costiveness from torpid bowels, we would remark that we never use purgatives for the purpose of affording relief, but rely on a wet bandage over the stomach and bowels; and occasionally dry-cupping becomes necessary. The whole alimentary canal, except the rectum, may be in a condition to respond to the stimulus of its contents, but in consequence of torpor of the rectum, no discharge takes place; in such cases, dry-cupping on the sacrum will oftentimes be followed by a discharge in less than an hour.

*Case* 9.—Roberts, (1849,) in his second year, troubled with cholera infantum, his teeth coming through very slowly. There appeared to be nothing dangerous in his case, and we tried the hydrarg cumcreta, followed by chalk julap; this afforded relief, temporarily, followed by a relapse two or three times. His parents had such unlimited confidence in our skill, that they did not send for any other physician on his third relapse, supposing that if we could not cure him, no one else could. Some weeks elapsed before we saw the child again, when we were summoned as a last chance, and found the little sufferer nearly dead. Pulse 120; tongue dry and furred; skin dry and shrivelled; extreme emaciation and prostration, while the discharges from the bowels threatened death in a day or two. While cogitating on his case, the remembrance of our own fate, and that of four brothers, came to mind:—we were all physicked until we could take no more physic, and when given up to die by the attending physician, as utterly beyond the reach of medicine, we all recovered, though we ourselves were paralyzed in the lower extremities, and our youngest brother was "laid out for dead;" we therefore concluded to let medicines go to Guinea, and try another plan. We had him immediately sponged from head to foot, in tepid water, to be continued twice daily, and a wet bandage, wrung out of tepid water, was applied over the stomach and bowels, covered with a dry one, to be renewed as often as it became too warm; and lastly, as a substitute for dry-cupping, we had a Cayenne pepper plaster applied the whole length of the spinal column, every night at first, and subsequently every alternate night, for the purpose of regulating the secretory system. The wet bandage soon cooled his fever down and relieved the irritability of the bowels, the sponging removed the dead epidermis and aided very materially in restoring the healthy action of the skin, while the pepper plasters restored healthy secretions and broke the chain of morbid nervous sympathy so completely, that the irritation from the gums could not be reflected on the ganglionic system; and finally, by judicious alternation of the diet, (sweet potato roasted in ashes, a little mutton suet warmed with a little loaf sugar, fresh butter without any salt, milk boiled with sweet gum bark in it, and soft boiled egg,) the little sufferer was soon relieved and permanently cured. Since that time, we have always, in such cases, endeavored to break the chain of nervous sympathy by pepper plasters, (never use mustard,) and relieve the irritability of the bowels by wet bandages; for young children, we have never used dry-cupping for fear of injury to the spinal column, while in its semi-cartilaginous state, but have used a Cayenne pepper plaster instead.

*Case* 10.—S. K., aged 75, very infirm, had been vomiting severely; all efforts to stop it had proved unavailing, and the stomach was so irritable it would bear neither water nor medicine of any description. Here was a puzzle. In ordinary cases, the vomitting could have been readily stopped by a cold wet bandage over the stomach, but the age of the patient, together with the extreme prostration induced by the incessant vomiting, rendered it highly probable that if cold applications should be made, reaction would never come on. Being familiar with the plan of reaching the abdominal organs through the spinal column, we had a compress about six inches square, wrung out of water as hot as the hands would bear, and applied to the lower cervical and upper dorsal vertebræ, and carefully covered with a dry one; the diagonal of the compress ranging with the spinal column. The hot, wet compress, frequently renewed, acted "like a charm" and checked the irritability of the stomach so much in the course of three or four hours, that a quilt could be slipped under him, and he was well sponged with hot water from head to foot; this started his skin into prompt, healthy action, and with a pleasant toddy or two he soon recovered.

*Case* 11.—Dr. Prather mentioned his own case to me. Having sprained his ankle by a fall from his horse, the pain in his back was as severe as in his ankle joint; and, with the hope of relieving the former, he had himself dry-cupped on the spine, and soon fell asleep. Next morning he felt quite comfortable, but was afraid to move his foot, for fear the intense pain of the evening previous would return, and actually held his foot motionless for several hours, but at last found there was very slight inconvenience from moving his foot; he consequently had himself dry-cupped regularly every alternate night, and recovered in one-third of the time usually required for such cases. During his convalescence *he noticed that his secretory system was performing its functions in a remarkably healthy manner*, and subsequent experience satisfied him that he could control the secretory system completely by dry-cupping the spinal column. The next day after he opened his office in St. Louis, a prominent citizen was thrown from his horse before the office and was carried in, and found to have a sprained ankle. Dr. P. treating him as he had treated himself, and effecting such a quick and comparatively painless cure, a large practice was immediately secured. By adding dry-cupping to his plans of treatment, he was enabled to cure chronic cases on which other physicians had exhausted their skill for fifteen or twenty years, and was overloaded with practice till his death.

*Case* 12.—Bob, a slave, having cut his leg severely with a broad-axe, we dressed the wound as usual, and as he appeared to be doing very well, we thought no more of the case. Boarding with his owner, we went out early next morning to see him, and was informed by his wife that he had not slept a wink the whole night. There being too many children in the cabin for him to sleep in the day time, we waited until night and then dry-cupped the whole length of the spine. He slept soundly that night, and next morning said his leg "did not hurt much." He was regularly dry-cupped every alternate night, and rapidly recovered, and to all inquiries concerning his leg, he invariably replied it did "not hurt much."

*Case* 13.—Mrs. L., called to see her on the third day after delivery; found her suffering most intensely; lochiæ suppressed, pulse 130, full and strong; dorsal decubitus, legs flexed, and every kind of bed clothing kept off the abdominal region, which was very hot and excessively tender; pain in the back very severe, skin hot and dry, tongue dry and furred; very considerable thirst; bowels did not need opening. An attendant was directed to stand by her bedside and re-apply, as soon as warm, a light cloth wrung out of moderately cold water, covering the whole abdominal region; her hands and face also to be frequently washed with cold water; this treatment to be kept up for four or five hours, then a quilt was to be slipped beneath her, and the whole surface, except the abdominal, sponged with warm water, and this finished, re-commence with the cold cloths to the abdominal region. To allay her thirst and produce diaphoresis, a pitcher full of cool water was prepared, with about twenty-five grains bi-carb. potass. to the pint, and she was directed to drink ad libitum. This solution was directed with a double view, to allay thirst and produce diaphoresis. The bi-carb. potass., though never classed as a diaphoretic, may be made to become one very readily; for water freely administered is by far the best diaphoretic we have ever yet been able to find, but it is attended with one disadvantage, the stomach will often refuse to tolerate enough, particularly if too cold. Now, in nearly every case of suppression of the action of the skin, there is an acid condition of the system, and by administering a solution of bi-carb. potass., twenty or thirty grains to the pint, the acid condition of the system can be easily removed, and, of course, all the disagreeable feelings resulting from such condition will be removed also; thirst allayed, and its weakening, harassing consequences removed, and, lastly, the stomach will tolerate water thus prepared in quantities sufficient to produce free, natural diaphoresis. On calling next morning found her tongue moist, abdominal tenderness much decreased—in fact could bear pressure without flinching, unless it was severe enough to reach the deep-seated organs. Being then able to turn on her side, she was dry-cupped the whole length of the spine, and the treatment continued as before, with the addition that the bowels were to be relieved at night by injections. Left her with the understanding that if she became worse notice was to be given immediately. Heard from her on the second day thereafter; fever was gone, lochiæ had returned, and she was occasionally walking across her room.

*Case* 14.—Mrs. H——n, (Logan county, Ky.,) suffering severely with pain in her back and limbs; skin hot and dry; left mamma much swollen, hot, dry, hard and glossy, pain in it very severe; tongue furred, and some dryness in the middle. She had been, accidentally, much exposed to the weather, and the above were the consequences. She was directed to be wrapped in a sheet, doubled and wrung out of water a little colder than the ordinary temperature of the body, and other covering suitably wrapped around her; if she fell asleep, not to be disturbed until she awoke; then a purgative dose of pil. hydrarg. and pulv. rhei was to be administered, and in the morning she was to be dry-cupped. She soon fell asleep and slept upwards of two hours, and having taken the purgative pills, rested tolerably well all night. She was sponged and dry-cupped next morning, the purgative acted finely, and she quickly recovered, without any additional visit.

*Case* 15.—Mrs. C. had been troubled with a running ulcer on her left arm for several months, during which time a physician had been trying to cure her. Directed her to apply the water dressing, sponge and dry-cup every alternate night, and having instructed her mother how to use a common tumbler, the directions were faithfully carried out, and at the expiration of three weeks, she showed me her arm smoothly healed over.

*Case* 16.—George, a slave belonging to Edward Campfield, Esq., Augusta, Ga., had suffered much for about four weeks from a distressing cough, during which time his mistress, who possessed a sound judgment, and had great experience in such matters, had exhausted her skill in preparing cough mixtures, but none afforded relief. Upon examination, found his pulse ranging from 120 to 130 every evening; skin dry, harsh and scaly; tongue furred and dry in the middle; coughing severely the whole night, with very short intermissions between the paroxysms. During the day the cough moderated much; the cervical and three upper dorsal vertebræ were excessively tender to the touch, demonstrating the correctness of the conclusion arrived at from auscultation, that the lungs and bronchiæ were not seriously involved, but that the cough was owing to spinal irritation. He was sponged every night, and dry-cupped every alternate night. In about two weeks, the pulse was reduced to the natural standard, cough much improved; but owing to his imprudence he at this time relapsed, and his pulse rose again to 120 for five or six days; a continuation, however, of the treatment for four or five weeks longer effected a cure.

*Case* 17.—Thomas M., (1848), a young man, had been afflicted with chills for six months, and the same physician mentioned in case 1st had been in attendance, but could not check the paroxysms more than from seven to fourteen days. Precisely the same treatment was pursued in this case as had been pursued by the old physician, with dry-cupping and sponging added thereto, and a cure was quickly effected. This case relapsed. We had it distinctly understood, as we always do when we undertake to cure a case of intermittent, that the patient is to abstain *in toto* from the use of milk; at the end of exactly two months, the patient took supper with his mother, and as he felt very stout and hearty, concluded a glassful of her nice, rich, cool milk certainly could not then hurt him, and he accordingly drank a glass of it. Next morning before ten o'clock he had a chill, and now, perfectly satisfied we were correct in forbidding its use, he promised if we would stop the chills once more he certainly would stand the temptation next time. We stopped them again by the same treatment, and for a period of four years, during which time we kept sight of the patient, he never had another chill.

*Case* 18.—Professor Prather, after having delivered a special lecture on dry-cupping one morning, remarked as we started to the hospital, that if there was a suitable case at the hospital he would demonstrate clearly the powerful influence of dry-cupping. The first case we reached was a man whose leg was swollen from the knee joint downward to about the ordinary size of a three gallon water bucket; it was extremely firm, uniformly solid, smooth and glossy; he had been to nearly every physician in St. Louis, and not one could make out the diagnosis, and Dr. P. himself confessed he could not make out a satisfactory diagnosis. Dr. P. directed that the man should keep his leg more in the horizontal position than he had been lately doing, and that he should be dry-cupped regularly. No other measure was adopted, and we were requested to watch the result of the case. At the end of ten or twelve days the absorbent vessels had been excited to such activity that the swelling was reduced in diameter to less than half the former size, and the diagnosis was then quite clear. The veins had become varicose, and so great had been the effusion and distension that the leg had become so uniformly hard as to render it impossible to tell what was the disease. Unfortunately, however, for our experiment, the man, finding himself so much improved, left the hospital, not being willing to remain the length of time necessary to effect a cure.

*Case* 19.—In the cholera year of 1849, for several weeks previous to the 9th of June, there had been very little sickness: about that time the first case of cholera occurred near Bowling Green, some fifteen miles distant, and in two or three days thereafter a truly "cholera atmosphere" prevailed, for not a single family escaped sickness; one of my neighbors had four cases of cholera morbus in his family one morning and three the next, and from that time until the middle of November every case to which we were called (except one surgical) was either vomiting or purging, and oftentimes both. The consequence was we had to ride night and day, and were in the saddle about sixty hours, without sleep, and to the great prostration caused by excessive fatigue, was added the derangement of the alimentary canal, caused by drinking a great variety of water. The skin ceased to act, and the kidneys did not secrete a drop of urine the whole day; about every half hour flashes of white light would dart through the optic nerves, followed immediately by excessive weakness and trembling, at the same time a violent vermicular motion would commence at the stomach and follow the convolutions of the bowels as far as the rectum; but in consequence of partial torpor of that part, and by dint of determined resolution the propensity to go to stool was stopped. These symptoms continued growing worse during the day, until late in the evening, when those flashes of weakness would come on it would be necessary to secure myself in my seat by holding to the horn of the saddle. During the whole day the kidneys were inactive, and all my disagreeable symptoms were attributed to the suppression of their action. When about two miles from home there was a profuse watery discharge from the bowels; but recollecting the case of a man in Russelville who saved himself during the first prevalence of cholera in that place, by firmly inserting a tampon of rolled newspaper into the rectum, we, having determined there should be no more discharges from the bowels, rolled the coat skirt so that the saddle would keep it firmly pressed against the rectum, and thereby restrain any further discharge. We reached home at sunset, and to relieve the distressing thirst and annoying sensations at the stomach, drank a pint of water with about thirty grains bi-carb. potass. dissolved in it, and in the course of an hour near another pint of the same solution; had the dry-cups applied the whole length of the spinal column; the first cup, at the junction of the cervical and dorsal vertebræ, could be felt, but the others could not. This torpor or partial paralysis of the spinal column, explained to me in my subsequent meditations why it was that the serous portion of the blood so readily transuded through the coats of the blood vessels into the alimentary canal, there becoming the well known "rice-water discharge;" and also explained how it was that the tampon of paper could be retained in the rectum and prove serviceable, instead of becoming such a source of irritation as to aid more in expelling than in retaining the contents of the bowels, as would ordinarily be the case. Having remarked to my wife that she would be a widow before the next night if the dry-cupping did not start the secretions, we dropped asleep and slept finely the whole night. On awakening next morning promptly put the hand to the forehead to ascertain the prospects of widowhood for my wife; found the forehead covered with a fine perspiration, and felt very comfortable.

While meditating on the disappointment our patients would experience at our failure to visit them that day we arose, and having walked across the room a few times, to our great surprise found our strength restored. After breakfast we started on our usual round, promising my wife to stop whenever my weakness should return, but, to our still greater surprise, the weakness did not return, and we actually rode thirty miles that day. The acid condition of the system, which so frequently follows the suppression of the action of the skin and the loss of a part of the serous portion of the blood, had been repaired by the absorption of the alkaline solution; the skin had yielded to the diaphoretic virtues of the water, and the kidneys, having been aroused from their torpor, had eliminated the poisonous morbific matter from the system, which so generally produces stupor or paralysis when they are torpid; and finally, all the secretory system being in full concurrent activity, the consequence was a remarkably speedy restoration to health. We took the hint thus accidentally given, and made it a standing rule to endeavor to restore the action of the skin, arouse to activity the whole secretory system, and freely use the bi-carb. potass., twenty or thirty grains to the pint, for a drink, and though, as before mentioned, every case (except a surgical one) to which we were called until the middle of November was either vomiting or purging, or both, yet we never lost a single case.

As we could not spare the time in many cases to dry-cup, we used a Cayenne pepper plaster three or four inches wide and long enough to reach the whole length of the spinal column as a substitute; in the milder cases we did not dry-cup or use the pepper plaster, being perfectly confident that the secretory system would resume its normal action just so soon as the skin should resume its natural action, and this we readily secured by frequent sponging and hot or cold wet bandages, and occasionally a wet sheet-pack, as circumstances required; these measures, together with the powerful diaphoretic virtues of water, rendered capable of toleration by the stomach by bi-carb. potass., rendered it easy to restore the patients to health. In many cases we used quinine. In those cases where it was necessary to relieve the bowels, we mostly used pil. hydrarg. and pulv. rhei and injections.

*Case* 20.—While examining, in conjunction with another physician, a lady attacked with cholera, we were requested to leave the room for a few minutes. We were not called in again for about ten minutes, and in that time so profuse had been the "rice-water discharges," that the patient's own mother would have hesitated to certify confidently concerning the identity of the patient, and the cramps which had been severe before were now general and agonizing. The other physician, having Dr. Cartwright's prescription of calomel, Cayenne pepper and charcoal ready in papers, a dose was immediately administered. As we had previously saved a patient in the collapsed stage by wrapping him in a blanket wrung out of water as hot as the hands could bear, we suggested it, and it was quickly tried, and in less than two minutes thereafter the cramping ceased entirely, and did not return. She fell asleep and remained in the wet blanket near three hours. In using this remedy, a dry towel should always be placed over the epigastric region. A pitcher full of moderately cool water was prepared with the bi-carb. potass. in solution, and she was allowed to drink as much and as often as she desired. The wet blanket was directed to be re-applied for an hour at bed-time, and a Cayenne pepper plaster the whole length of the spinal column. The whole secretory system was aroused to healthy activity, and she recovered without further medical attendance.

It is quite likely the other physician attributed the cure to Dr. Cartwright's prescription, but we are quite confidant that nothing else under the sun would have relieved the cramping in two minutes, but the hot wet blanket. The prescription of Dr. C. had been administered twenty or thirty minutes previously, without producing any relief whatever.

*Case* 21.—James Barrett, Augusta, Ga., Fourth Georgia regiment, letter of company forgotten—but the fact can be substantiated by at least fifty other residents of this place—a soldier discharged for paralysis arising from the severing of the median nerve. His arm was so utterly useless to him, that in order to keep it from dangling continually in his way, he carried it in a sling; it was much shrunken, cold and blueish; fingers bent and stiff; could not touch a single finger with his thumb. We told him we thought he could be cured, and volunteered to do it free of charge; and accordingly dry-cupped him for about three months, making him take his arm out of the sling, so that the circulation could be carried on better, at the commencement of the treatment. At the expiration of that time, he could touch any finger with his thumb, and could grasp his musket with his once paralyzed hand, and lift it with ease from the ground. We did not continue the dry-cupping as long as we desired, owing to some alteration in his arrangements; he, however, continued to improve, and finally re-volunteered in the army, and is said now to be in active service. If there is any other remedial agent which would have effected a cure, we have never been able to read or hear of it.

We now propose to drop the dogmatic affirmation formerly necessary, and by the clear expositions of the excito-secretory system, furnished us by Dr. H. F. Campbell, we can give a clear and logical rationale of the treatment above referred to, and we invite a close, logical scrutiny, so that the reader, if satisfied of its correctness, will be prepared to act accordingly, justly concluding that any "hobby" capable of accomplishing so much is well worth the riding. We shall quote a few pages from "The Secretory and the Excito-Secretory System," by Dr. H. F. Campbell. Page 94, "This cerebro-spinal system has been subdivided into two portions, viz: the nerves of sensation and the nerves presiding over muscular action. A *relation* has been discovered to exist between these two portions of the nervous system, by virtue of which the sensory nerves have been found to act as excitors to the motory; and hence this system has been termed by Dr. Marshall Hall the excito-motory system of nerves.

"Now, as we hope more fully to set forth in the present discussion, these same sensory nerves are not only excitors to the *motory* system, but, under certain circumstances, most of them sustain an analogous relation to the *secretory* nerves, exciting them and modifying their action, diminishing, increasing and altering the secretions, according to the extent and character of the excitation applied. It will be our object, then, to show that the sensory nerves, or at least some of them, sustain to the other two portions of the nervous system a *double* relation; first, excitors to the motory system giving rise to the excito-motory system described by Dr. Marshall Hall in 1837; and, secondly, excitors to the *secretory* system, resulting in the excito-secretory system enunciated first in this country in the year 1850. * * * * * * P. 116. We have seen that inflammation, pain, and irritation are produced *locally* by the process of dentition evinced by restlessness, biting, &c. Secondly, we have seen that this local irritation can be transmitted by excito motory influence to other and distant parts of the body, manifested by convulsions. We have also endeavored to corroborate this latter opinion by a reference to the order of succession in the nerves in which this irritation occurs, and also by a comparison of these phenomena with other well understood and established analogous phenomena. Heretofore we have had to deal entirely with functions of the cerebro-spinal system of nerves; but to account for this second and more obscure part of our problem, we must look in vain to any direct anatomical connection between the fifth pair and the rest of this system of nerves. *We are forced to seek out other connections, indeed somewhat more intricate and indirect, but fortunately no less legitimate and definable.* We have now to consider a set of organs which, unlike the voluntary muscles, have no connection, or rather, we would say emphatically they have a connection, though indirectly, with the cerebro-spinal system. We mean the abdominal viscera, which we know are almost altogether supplied from the great sympathetic system of nerves. *Now, in the prosecution of our inquiry, it becomes necessary to the elucidation of the question to trace out the same connection between the fifth pair and the sympathetic or secretory, as we did between the fifth pair and the cerebro-spinal or motory nerves,* and then, should we succeed, we will briefly inquire into the bearing which this connection and its possible results may have upon our question.

"The connections between the fifth pair, the rest of the cerebro-spinal system of nerves, and the great sympathetic, are so abundant and universal that it is only necessary to enumerate a few of them. First, we have a connection in the ophthalmic or first division by its nasal branch, communicating with the ciliary ganglion; then in the superior maxillary or second division are branches of communication with Meckel's ganglion; again, in the sub-maxillary ganglion, with the inferior maxillary or third division. So much for the fifth itself. Then we know that every one of the spinal nerves throughout the entire cord are connected to each sympathetic ganglion of the system, thus establishing communications the most abundant and intimate between these two systems of nerves. We know also that these ganglia distribute numerous branches to all the splanchnic viscera by plexuses which accompany the arterial trunks into the minute structure of these organs.

"Thus connected and distributed, this nerve presides over the important functions of nutrition and secretion, which office so characterizes it as to give it the name of the SECRETORY system. In the physiology of the nervous system, there is no fact better established by anatomy and pathology, as well as by experiments on the lower animals, than this—

that the sympathetic nerve, whatever else may be its function, always forms a necessary element in the nutrient and secretory apparatus of all the splanchnic viscera; and further, that upon its sanity depends the due administration of these two great functions. It is the nerve for the blood vessels, 'and,' remark Todd & Bowman, 'as secretion is mainly dependant on the normal nutrition of glands, it is reasonable to suppose that function would be to a certain extent controlled by these nerves.'

"(Dr. Carpenter, in speaking of the functions of the *true* sympathetic fibres, says, 'There appears strong grounds for the conclusion that the office of these fibres is to produce a direct influence upon the chemico-vital processes concerned in the organic functions of nutrition, secretion, &c.; an influence which, although not essential to the performance of each separate act, may yet be required to harmonize them all together, and to bring them into connection with mental states." Principles of Human Physiology, 1853, page 830.) Page 47—Sir Charles Bell's opinion is somewhat similar. 'We are left to the conjecture that the sympathetic or ganglionic system of nerves, according to Bichat, are for those thousand secret operations of a living body which may be called constitutional; circulation, secretion and absorption are operations which simultaneously affect the entire frame.' Page 118—"And as early as 1732, Pourfour du Petit found that the division of the trunk of the sympathetic, opposite the fourth or fifth cervical vertebra in dogs, was followed very rapidly by great disturbance of the circulation of the eye-ball, producing inflammation, flattening of the cornea, and finally destruction of this organ." Page 50—The experiments of Dupuy upon horses, wherein he extirpated the superior cervical ganglion, were followed by the same results with regard to the local effect in the eye, but also with the more apposite and corroborative consequences, that there was *an eruption over the whole cutaneous surface*, with emaciation and an œdematous state of the limbs." "Dr. John Reid has also experimented on the sympathetic nerve in the neck, and found the eye similarly affected with the above, the conjunctiva becoming red and congested in a few minutes, while in other experiments the eye presented an ecchymosed or bloodshotten appearance." Page 118—We have now glanced sufficiently, we think, at the anatomy and physiology of the sympathetic system of nerves, to make the application of such points as are pertinent in the solution of our pathological problem. In its anatomy, we have seen its connections with all three of the divisions of the fifth nerve by ganglia, the connection of these various ganglia with each other, as well as with the cerebro-spinal axis; and lastly, the distribution of branches from these ganglia, which are conducted by the arteries into every part of every one of the splanchnic viscera. In its physiology, we find it in entire charge of the important functions of nutrition and secretion, and that, wherever these processes are effected, it is by the agency of this nerve alone upon the blood-vessels. And further, that pathology and experiments on lower animals establish its indispensableness to the due performance of these functions, and that, whenever the supply of its innervation has been cut off from a part of the organism, that part immediately manifests symptoms of impaired nutrition and altered secretion. Now, we are all aware that nearly the whole of the intestinal canal, or rather that portion between the stomach and lower part of the colon, receives no direct innervation from the cerebro spinal axis, but is entirely dependent upon the sympathetic nerve for its supply of nervous influence of whatever kind it may enjoy, whether motory, sensory, or secretory, and consequently an impairment of the function of this nerve must necessarily correspondently alter its condition, so far as regards all those functions with which this nerve endows it. The alterations in these functions would of course depend in a great degree upon the amount of impairment in the source of irritation; thus, as we have seen, if the supply is entirely cut off, the functions of the arteries seem in a great measure to cease, passive congestions occur and the parts inflame and ulcerate. Now, we can also very naturally conceive of a condition of these nerves somewhat analogous to the above, yet intermediate between the entire interruption caused by section and perfect health—a condition of embarrassed or exalted innervation. Now, this intermediate condition is exactly the state which, from the developments of the foregoing investigation, we feel that we are authorized to affirm is that which occurs in severe dentition, and that upon it is dependent the whole train of intestinal morbid phenomena observable during this process. That this, so far, is legitimately inferable, we do not think any one will deny. Now let us inquire how far these phenomena are dependent upon dentition, and *analogy*, with the *excito-motory* system, will much assist our argument.

"We have seen that local irritations can, through this system, produce convulsions by the reflex function of the nerves, the sensitive branches of the fifth pair becoming excitor to the motory spinal nerves; and so may we justly infer do these same branches, under certain circumstances, become excitor to the *secretory* filaments of the sympathetic, distributed so abundantly to the intestinal canal, by transmission of this irritation through the various ganglia with which it is connected. Thus the irritation at first produces simply an exaltation of the innervation of these secretory surfaces, and consequently secretion is more active than normal, producing simple *diarrhœa*. A continuance of the irritation alters the character of the secretion, and we have the various morbid discharges observable during this period. This increase and change in the secretion are effected by the agency of the altered function of the nerve upon the arteries from which these secretions are eliminated. Now, when the innervation of these arteries is still further embarrassed by the long continuance of the *reflected irritation*, the state of things nearly approaches that observed in Dupuy's, Reid's and Pourfour du Petit's experiments of actual destruction of the nerve, and we have ulceration of the intestinal mucous membrane;—all these phenomena being the result of various degrees of injury sustained by the sympathetic nerve. * * * * P. 121. In conclusion, let us define the position which, at the end of our investigation, we feel warranted in assuming. It is the following: that in the anatomy and physiology as well as in the dependent analogies of the process of dentition, we find ample ground for the opinion that the diseases pertaining to this period *may be dependent, and in many cases are entirely so, upon the local irritation attending the process being transmitted either through the cerebro-spinal system of nerves, producing convulsive diseases in the motory apparatus, or through the sympathetic, causing derangements in the secretory organs, particularly the alimentary canal, by the sway which it exercises over the arterial system, from which these secretions are eliminated.* And the practical deductions to be drawn from these conclusions are, that we should not be remiss in taking every measure to arrest or lessen this local irritation, either by free and repeated incisions of the gums, or by the judicious administration of appropriate remedies, among which we have found opiates prove most safe and efficient."

We would beg leave to suggest, with all due deference to Dr. C.'s superior skill, that the "practical conclusions" we would draw from the premises before us, would be to *break the chain of morbid nervous sympathy, and keep it broken by dry-cupping or its substitute,* (the same remedy serving at the same time to bring the secretions back to the healthy standard,) and to relieve the irritation, inflammation or ulceration already excited in the alimentary canal by the long continuance of vitiated secretions, by hot or moderately cold, wet bandages to the abdominal region; and, at the same time, pay due attention to the skin, a torpid condition of which is alone amply sufficient to become an excitor to the secretory system, and cause it to pour out profuse or vitiated secretions; then, with judicious management of the diet, we may reasonably expect a cure in a far greater number of cases than we possibly can when such measures are neglected.

The flood of light thrown on the subject of the excito-secretory systems, contained in the above quotations of Dr. Campbell's masterly work, shows us plainly now, how the whole secretory system can be reached readily, certainly and normally, by dry-cupping on the spinal column, and it demonstrates that the dogmatic affirmation of Dr. Prather, that he could control all the secretions with certainty and efficiency by that measure, was in strict harmony with physiological laws, and it leaves all opponents without a shadow of an excuse for neglecting such a powerful remedial agent. Such is its power over the secretory system that we twice produced bilious diarrhœa, by having our own spinal column dry-cupped the whole length, at the first trial; and in two other cases same results were produced—the liver responded so readily that a profuse secretion of bile was poured out, and bilious diarrhœa could not be avoided; after about half a dozen discharges the diarrhœa in each case ceased. Another case fell under our notice where injurious effects followed. A physician, to whom Dr. Prather recommended dry-cupping, forthwith tried it on a patient afflicted with chorea, and the next day the nervous agitation was worse. These cases led us to adopt the plan of gently dry-cupping at first, only using two cups, and gradually increasing the number; and since that time, no injurious effects whatever have been observed. If, however, the patient is suffering from severe pain, or a wound, it may be tried the whole length at first.

We now propose to present certain propositions which we consider to be fairly deducible from all the data above presented, and to their consideration we invite the reader's calm, close and logical scrutiny, for many tens of thousands of persons have died prematurely, since Dr. Prather first made his discovery, in consequence of the heedless, thoughtless derision of "Prather's hobby."

We candidly think Dr. Campbell has made the subject so plain, that no reader can really consider himself excusable *in foro conscientiæ*, if he shall in future neglect such a powerful means of saving life and relieving suffering; and we therefore invite the most logical scrutiny, presuming no one would be willing to act, unless satisfied of the correctness of his course.

From the above cases and expositions, taken collectively, we may safely affirm—

*Proposition* 1.—That dry-cupping on the spinal column, by means of the intimate connection of the cerebro-spinal nerves, with the ganglionic system, is a powerful excito-secretory remedial agent.

*Proposition* 2.—That its proper and useful effects are to bring the performance of the functions of all the various organs to the normal healthy standard.

From Case 9 we may deduce

*Proposition* 3.—That when the secretions are already in excess, as its uniform effects being the bringing of all the organs to perform their functions in a proper healthy manner, it becomes a sedato-secretory agent by breaking the chain of morbid nervous sympathy, thereby cutting off the irritation which had produced that excess of secretion.

From Cases 1, 4 and 18 we may deduce

*Proposition* 4.—That dry-cupping is a powerful excitant of the absorbent system

From nearly all the cases we have

*Proposition* 5.—That it is the most powerful and useful tonic and alterative agent now known to the profession.

From Cases 11 and 12 we have

*Proposition* 6.—That by breaking the chain of morbid nervous sympathy, it may become an anodyne.

From Case 6 we have

*Proposition* 7.—From its effects in breaking the chain of nervous sympathy in cases of pregnancy, preventing most of the usual disagreeable consequences of that condition, we may style it an anticipating or proleptic anodyne.

From Case 7 we have

*Proposition* 8 —That it is the best remedy now known to break the chain of nervous sympathy in pregnancy and prevent habitual miscarriage.

From Case 5 we have

*Proposition* 9.—That it is the best remedy now known to shorten tedious labors.

From Case 5 we have, also,

*Proposition* 10.—That it is the best remedy now known to take off muscular resistance in the reduction of dislocated joints, and also the muscular resistance of the urethra, when it becomes necessary to insert a bougie or catheter, or to cure chordee.

From Case 5 we have

*Proposition* 11.—That from its producing such opposite effects as contraction of the fundus uteri and relaxation of the os uteri at the same time, we have an indisputable proof of the correctness of Proposition 2

From the experiments of Dupuy upon horses, wherein the extirpation of the cervical ganglion was followed by a *cutaneous eruption over the whole surface*, we have

*Proposition* 12.—That disease of the ganglionic system may often be unnoticed, and only manifest itself by severe cutaneous affections.

From Cases 1 and 2 we have

*Proposition* 13.—That if, in most cases of severe cutaneous affections, instead of making local applications to the part affected, we would proceed upon the assumption that the cutaneous disease was the effect of a cause operating through the ganglionic system, and restore that system to its healthy action by dry-cupping, we should certainly effect a cure, as was effected in those cases.

From the thousands of cases of diarrhœa, dysentery and cholera morbus constantly occurring, we have

*Proposition* 14.—That a suppression of the action of the skin is amply sufficient to act as an excito secretory cause, developing one or the other of those diseases, and that, if we remove the cause by restoring the normal action of the skin, we can cure such cases far more readily than by relying upon opiates, astringents, mercurials or purgatives, and neglecting the skin, as too many now do.

From Case 9 we have

*Proposition* 15.—That a vast proportion of the cases of soldiers discharged by reason of chronic diarrhœa and dysentery, may be readily cured by regulating the secretions by dry-cupping, keeping up the healthy action of the skin by frequent sponging, and relieving the irritability, inflammation or ulceration of the alimentary canal, induced by the deranged condition of the ganglionic system, by a wet bandage, hot or cold, as circumstances may require.

From hospital statistics and the results of the ordinary practice we have

*Proposition* 16.—That a large proportion of such cases of chronic diarrhœa and dysentery *cannot be cured* by the ordinary plans of treatment.

From Dr. Campbell's proposition, (page 119,) that "local irritation can, through this system, produce convulsions by the reflex functions of the nerves, the *sensitive branches* becoming *excitor* to the motory spinal nerves;" and so, we may justly infer, that these branches, under certain circumstances, become excitor to the *secretory* filaments of the sympathetic—we have

*Proposition* 17.—We may justly infer, in traumatic cases, that the irritation of the wound becomes excitor to the secretory filaments of the sympathetic; hence we have either wastings, continued fever and profuse discharge of pus from the wound, or vitiated secretions and morbid discharges from the lungs or bowels.

As we have demonstrated, by successful practice, that the chain of morbid nervous sympathy, in cases of dentition, can be broken, and the ordinary evil consequences of such irritation be prevented, and also in Cases 11 and 12, we therefore deduce

*Proposition* 18.—That, in traumatic cases, the chain of morbid nervous sympathy can be broken and kept broken by dry-cupping, thereby preventing the usual evil consequences of such irritation.

In cases of dentition, when the irritation has continued long enough to produce actual and serious disease of the alimentary canal, breaking the chain of nervous sympathy extending from the gum to the alimentary canal, will not alone be sufficient to effect a cure, it being necessary also to relieve the irritability of the alimentary canal by wet bandages; we therefore deduce

*Proposition* 19.—That likewise, in traumatic cases, breaking the chain of nervous sympathy by dry-cupping will not alone be sufficient, but it will be necessary to relieve the irritation in the wound itself, or in the part to which it may have been reflected, by the water-dressing, and prevent the skin from becoming an excito-secretory agent through the suppression of its action, by frequent sponging of the entire surface.

*Purely Theoretical.*

From the prompt relief afforded in the two cases of cramping in the collapsed stage of cholera, by wrapping the patients in blankets wrung out of hot water, thereby stopping the cramping in two minutes, we may logically deduce

*Proposition* 20.—That the diseased condition of the skin had become an excitor to the motory spinal nerves, bringing on cramps.

From the well-known fact that wounded soldiers who are left on the field of battle during cold nights, are far more likely to be affected with tetanus, and that idiopathic tetanus occurs, we may deduce

*Proposition* 21.—That in cases of traumatic and idiopathic tetanus, the skin has become, as in the cramping stage of cholera, an excitor to the spinal motory nerves.

From the fact that the patients in the collapsed cramping stage of cholera were immediately relieved by enveloping them in hot, wet blankets, we have

*Proposition* 22.—That in cases of traumatic or idiopathic tetanus, we may relieve the deranged condition of the spinal motory system, by likewise waapping the patient in a hot, wet blanket, and by keeping the chain of morbid nervous sympathy broken, and all the organs in healthy action by dry-cupping, a cure can be effected.

Let us quote again from Dr. Campbell, page 50–1:

"The experiments of Dupuy upon horses, wherein he extirpated the superior cervical ganglion, were followed by the same results with regard to the local effect in the eye, but also with the more apposite and corroborative consequences, that there was an eruption over the whole cutaneous surface, with emaciation and an œdematous state of the limbs. Dr. John Reid has al-o experimented on the sympathetic nerve in the neck, and found the eye similarly affected with the above, the *conjunctiva becoming red* and congested in a few minutes, while in other experiments the eye presented an *ecchymosed or bloodshotten appearance.* * * * * * * Now, a reference to some of the pathological phenomena of typhoid fever will discover a close analogy, if not identity, to the above results. In the first place, the conjunctival congestion—its character, the attendant suffusion, together with the entire freedom from pain, even on exposure to the strongest light; while, at the same time, none of the symptoms of true inflammation are present; all indicate the seat of the nervous derangement upon which it depends, to be the ganglionic system, and not the cerebro-spinal, the analogous arrangements of which are invariably of a sthenic character, and attended with acute pain in the region in which they occur Again, an attentive consideration of the character of these congestions will show that it does not vary in any respect, except in degree, whether occurring in the mucous membrane of the eye, that of the stomach, pharynx, small intestine, large intestine or bladder in the typhoid type, or on the cutaneous surface in the typhus. In all the above localities, and under all circumstances, we find the capillary congestions wearing the same aspect, assuming invariably a *passive* character, often approaching the condition of true stasis, but never attended with the florid redness, the pain or the swelling of active inflammation. Lastly, in the cutaneous petechial eruptions or masculæ of the typous type of continued fever, we can also detect the same character of *passive congestion* from deficiency of nervous energy carried to a still greater degree. In this type the nervous power of the cutaneous capillaries is so far diminished, that it amounts to a state of actual paralysis, allowing such distension of the capillaries that their rupture and a sub-cutaneous effusion is the result"

From Case 4, wherein it is shown that the suppression of the action of the skin developed itself by involving the ganglionic system, producing inflammation and a profuse discharge of pus from the inflamed part, we have

*Proposition* 23.—That suppression of the action of the skin may seriously involve the ganglionic system and all parts dependent thereon for nervous influence.

And conversely, from Cases 1 and 2, we have

*Proposition* 24.—That serious disease of the ganglionic system may keep up disease of the skin, or in other cases keep up torpor of the skin.

And from both these propositions we deduce

*Proposition* 25.—That the great obstinacy of typhus and typhoid fevers is owing to severe disease both of the skin and the ganglionic system, they mutually re-acting on each other, and each preventing the other from resuming its natural, healthy action.

And lastly, from all the data before us, we have

*Proposition* 26.—That, in typhus and typhoid fevers, a frequent wet sheet pack, wrung out of water about ordinary temperature of the body, would be a powerful adjuvant to reduce the pulse and fever heat, and restore the action of the skin; that a wet bandage, covered with a dry one, laid over the whole body during the interval between the wet sheet pack, would answer admirably to relieve the irritability or inflammation of the alimentary canal, produced by the conjoint derangement of the skin and the ganglionic system, while diaphoresis is produced by the free use of water and the bi-carb. potass.; and lastly, that dry cupping, in consequence of being a powerful excito-secretory remedial agent, would cause all the various organs to perform their functions in a healthy manner, thereby soon removing the main causes of these diseases, and proportionally shortening their duration.

In conclusion, we would most earnestly urge the above propositions upon all interested, for a more powerful and useful remedial agent than that discovered by Dr. Prather, and the rationale of whose modus operandi has been so ably elucidated by Dr. Campbell, has never yet been conferred on man; and the welfare of thousands of our soldiers, who have so nobly sacrificed themselves in our glorious cause, demands that the profession should cease to deride it as a "hobby," and should take it up and test its effects impartially. If any surgeon desires to criticize this article, all we ask is, that he shall select a number of patients wasting away from a profuse discharge of pus, and having applied the water dressing to the wound, and to any other part to which the irritation may have been reflected, then sponge and dry-cup every alternate night for one month, and then criticize as he may deem most advisable.

One peculiarity concerning it and we have done. It produces its best effects when the patient can sleep or retain the recumbent position for some hours after the operation.

To the calm, candid and conscientious investigator we cheerfully submit the foregoing.

